# Accuracy of methionine-PET in predicting the efficacy of heavy-particle therapy on primary adenoid cystic carcinomas of the head and neck

**DOI:** 10.1186/1748-717X-8-143

**Published:** 2013-06-13

**Authors:** Sachiko Toubaru, Kyosan Yoshikawa, Seiya Ohashi, Katsuyuki Tanimoto, Azusa Hasegawa, Koji Kawaguchi, Tsuneo Saga, Tadashi Kamada

**Affiliations:** 1 Research Center for Charged Particle Therapy, National Institute of Radiological Sciences, 4-9-1 Anagawa, Inage-ku, Chiba 263-8555, Japan; 2 Oral and Maxillofacial Surgery, Tsurumi University School of Dental Medicine, 2-1-3 Tsurumi, Tsurumi-ku, Yokohama 230-8501, Japan

**Keywords:** C-11-Methionine PET, Carbon ion radiotherapy, Head and neck adenoid cystic carcinoma, Univariate analysis, Multivariate analysis

## Abstract

**Background:**

We evaluated whether or not PET or PET/CT using L-methyl-[11C]-methionine (MET) can allow for the early prediction of local recurrence and metastasis, as well as the prognosis (disease-specific survival), in patients with adenoid cystic carcinoma of the head and neck treated by carbon ion beam radiotherapy.

**Methods:**

This was a retrospective cohort study of sixty-seven patients who underwent a MET-PET or PET/CT study prior to and one month after the completion of carbon ion radiotherapy (CIRT). The minimum follow-up period for survivors was 12 months. The MET accumulation of the tumor was evaluated using the semiquantitative tumor to normal tissue ratio (TNR). A univariate analysis was conducted using the log-rank method, and the Cox model was used in a multivariate survival regression analysis.

**Results:**

The average TNR prior to and following treatment was 4.8 (±1.5) and 3.0 (±1.3), respectively, showing a significant decrease following treatment. In the univariate analysis, a high TNR prior to treatment (TNRpre) was a significant factor for predicting the occurrence of metastasis and the disease-specific survival. A high TNR following treatment (TNRpost) was a significant factor for predicting the development of local recurrence. The residual ratio of TNR changes (TNRratio) seemed to be less useful than the TNRpre. In the multivariate analysis, the TNRpost and tumor size were the factors found to significantly influence the risk of local recurrence. The TNRpre, TNRratio and tumor size were all significant factors influencing the occurrence of metastasis. Regarding the disease-specific survival, the TNRpre and age were the only factors with a significant influence on the outcome.

**Conclusions:**

The TNRpre was a factor that was significantly related to the occurrence of metastasis and the disease-specific survival after CIRT for adenoid cystic carcinoma of the head and neck. The TNRpost was a factor that was significantly related to the development of local recurrence. Thus, MET-PET or PET/CT can be useful for predicting or determining the therapeutic efficacy of CIRT.

## Background

Adenoid cystic carcinomas are malignant tumors generated from the secretory epithelial cells of the salivary glands, accounting for about 10% of all salivary gland tumors. They are relatively rare tumors, representing less than 1% of all malignant tumors in the head and neck region [1,2]. Although their development is slow, they have a strong tendency for local invasion and locally recur at a high frequency. They are also characterized by the frequent occurrence of metastasis during the clinical course over a long period of time [2-6]. Radical excision by surgery has been the primary treatment option, however, wide-range excision can be difficult due to the complicated anatomical structure in the head and neck region. A decrease in quality of life following surgery is also unavoidable due to the loss of oral functions and aesthetic issues due to formal changes in the tissues. Adenoid cystic carcinomas have low radiation sensitivity, and the local control rate is unsatisfactory with radiation therapy using conventional X-rays [7]. Furthermore, there has been no clear evidence regarding the effects of chemotherapy [4,8].

### Heavy-particle therapy

Carbon ions are a type of heavy ion associated with high linear energy transfer (LET) radiation with high relative biological effectiveness (RBE). The biological characteristics of these ions include that there is little recovery observed following sub-lethal damage (SLD) [9], and smaller differences in sensitivity due to the phase in the cell cycle [10] and due to the oxygen concentration (low OER: oxygen enhancement ratio) [11] compared to low LET radiation, such as X-rays or proton beams. The physical characteristics of these carbon ions include their having a high relative dose, called a Bragg peak, which reaches about 15 cm deep from the body surface. Consequently, carbon ions have excellent efficacy when used in focused dose concentrations, in addition to allowing for treatment at a good dose distribution with fewer side effects on normal tissues [12]. Taking these characteristics into consideration, it is believed that carbon ions can also provide an effective therapeutic method for cancers that are resistant to conventional radiation.

### L-methyl [C-11]-methionine (MET)

The amino acid metabolism in malignant cells is related to various catabolic processes associated with tumor growth [13]. MET is a neutral essential amino acid that plays a key role in cancer cell metabolism [14]. MET is a necessary amino acid for protein and polyamine synthesis, as well as transmethylation reactions. Because of their enhanced levels of these reactions, the uptake of MET into cancer cells is enhanced [15-17]. This metabolism can be visualized by positron emission tomography (PET) using radiolabeled MET. However, the use of MET imaging is restricted to PET centers with an in-house cyclotron and radiochemistry facility because of the short half-life (20 min) of C-11.

### Carbon ion radiotherapy and PET for head and neck cancers

In our institute, a Phase I/II carbon ion radiotherapy (CIRT) trial has been conducted in patients with unresectable head and neck cancers, and 669 head and neck tumors had been treated with CIRT as of March 2010 [18-20]. The three-year local control rate of adenoid cystic carcinomas of the head and neck in patients treated with carbon ion radiotherapy (CIRT) was 82%, and the five-year survival rate was 68% [19,20]. A MET-PET (or PET/CT) study was performed to evaluate the tumor prior to and following heavy-particle therapy. In bone and soft tissue sarcomas and chordomas, we have reported that the development of local recurrence could be predicted using the rate of decrease in MET accumulation prior to and following heavy-particle therapy [21,22]. In choroidal malignant melanomas, although there were no changes in the long-term tumor size observed in MRI studies following heavy-particle therapy, decreases in MET accumulation were observed in most cases from six months or more after treatment [23]. The usefulness of MET-PET in evaluating heavy-particle therapy for adenocarcinoma of the head and neck has previously been examined [24]. The present study was a retrospective cohort study to investigate the accuracy of MET-PET (or PET/CT) for predicting the efficacy of heavy-particle therapy against primary adenoid cystic carcinomas of the head and neck.

## Methods

### Patients

CIRT for head and neck tumors is used in patients for whom a surgical resection is difficult, or for patients who reject surgery and wish to undergo CIRT. Patients who had undergone chemotherapy within four weeks prior to CIRT and for whom radiotherapy had already been performed at the tumor site were not eligible for CIRT [19]. In this study, we included only the patients who were histologically diagnosed to have primary adenoid cystic carcinoma and for whom CIRT was performed. All of the patients in this study received MET-PET or PET/CT examinations both prior to and following CIRT. We excluded patients who had received chemotherapy prior to CIRT, even if it was more than four weeks prior, from this study. Patients whose cause of death was unknown were also excluded from this study. The institutional ethics committee (National Institute of Radiological Sciences) approved the study, and written informed consent was obtained. There were very few patients with adenoid cystic carcinomas of the head and neck, so it took a long time to collect a sufficient number of cases. There were 67 primary cases that received CIRT during the period between October 1995 and December 2009, and that underwent a MET-PET (or PET/CT) study prior to and following treatment. The subjects consisted of 27 males and 40 females, with an average age of 54 ± 15 years old (15–84 years old). Patients were staged according to the fourth edition of the TNM staging system (International Union Against Cancer; UICC, 1987) [25], and there was one case of T1 (2%), six cases of T2 (9%), nine cases of T3 (13%) and 51 cases of T4 (76%) disease. The average follow-up period following treatment was 50.8 ± 33.8 months (10–180 months), and the minimum follow-up period for survivors was 12 months. The first follow-up examinations, including MET-PET, CT and MRI, were performed four weeks after the completion of CIRT. Further CT or MRI studies followed every two or three months for the first two years and every four to six months for the rest of the follow-up period. The tumors were evaluated according to the World Health Organization definitions [26]. The development of metastasis and local recurrence during the follow-up period were diagnosed based on the imaging information obtained by periodic CT or MRI showing an obvious increase in the tumor or nodule size, i.e. progressive disease was defined as a more than 25% increase in the product of the perpendicular diameters of the tumor. A summary of the cases is provided in Table 1.

**Table 1:** Patient characteristics (n = 67 patients)

**Characteristic**	**No. of patients**	**% of patients**
**Gender**		
Male	27	40%
Female	40	60%
**Age**		
10-19	1	2%
20-29	3	4%
30-39	7	10%
40-49	14	21%
50-59	18	27%
60-69	15	22%
70-79	8	12%
80-89	1	2%
**Tumor site**		
Nasal cavity	7	10%
Paranasal sinus	21	31%
Salivary gland	9	13%
Oral cavity	14	21%
Pharyngolarynx	10	15%
Orbita	2	3%
Lacrimal gland	2	3%
Lacrimal sac	1	2%
Ear canal	1	2%
**Tumor size in mm**		
≤40	20	30%
>40	47	70%
**T Stage**		
1	1	2%
2	6	9%
3	9	13%
4	51	76%

### Carbon ion radiotherapy protocol

A heavy particle accelerator system and the biophysical characteristics of the carbon ion beam have been described in a previous report [12]. The protocol used for CIRT has also been described in detail in a previous report [19]. CIRT for malignant tumors in the head and neck region is targeted at advanced cases in which surgical excision is determined to be difficult, or cases in which the patients refuse surgery and prefer CIRT. The cases in which chemotherapy was performed within four weeks prior to treatment, or in which radiation therapy had already been performed at the treatment site were excluded from the indications for treatment. Table 2 shows the regimen of CIRT used in this study.

**Table 2:** Carbon ion radiotherapy regimens

**Tumor site**	**Number of patients**		**Total dose (GyE)**	**Fraction dose (GyE)**
Nasal cavity	7	(10%)	52.8-70.8	3.3-4.4
Paranasal sinus	21	(31%)	52.8-70.8	3.3-4.4
Salivary gland	9	(13%)	57.6-64.0	3.6-4.0
Oral cavity	14	(21%)	57.6-64.0	3.6-4.1
Pharyngolarynx	10	(15%)	57.6-64.8	3.6-4.0
Orbita	2	(3%)	57.6, 64.0	3.6, 4.0
Lacrimal gland	2	(3%)	57.6, 64.0	3.6, 4.0
Lacrimal sac	1	(2%)	57.6	57.6
Ear canal	1	(2%)	52.8	52.8

### PET and PET/CT studies

MET with a high specific activity was produced by the standard technique using a method modified from the synthesis reported by Langstrom *et al.*[27]. The MET studies for adenoid cystic carcinomas of the head and neck were carried out with a PET device over the period between October 1995 and September 2007. The studies were changed to a PET/CT device, and patients were examined using the new device between January 2003 and February 2010. In the PET study, whole-body scanners (ECAT EXACT HR+ and ECAT EXACT 47; Siemens CTI, Knoxville, TN, USA) were used. In the PET/CT study, whole-body PET/CT scanners (Biograph duo and Biograph 16; Siemens CTI, Knoxville, TN and Aquiduo; Toshiba Medical Systems Corporation, Otawara-shi, Tochigi-ken) were used. The ECAT EXACT HR+, ECAT EXACT 47, Biograph duo, Biograph 16 and Aquiduo were used to scan 29, 9, 10, 7 and 12 patients, respectively. The same PET device as was used for the MET-PET study prior to the treatment was also used to carry out the study following the treatment so that the results could be easily compared. With the ECATs and Biograph duo, emission data corrected for random events, dead time and attenuation were reconstructed by filtered backprojection using a Ramp filter with a cut-off frequency of 0.4, followed by decay correction. With the Biograph 16 and Aquiduo instruments, emission data with the corrections were reconstructed by OSEM using 16 subsets, four iterations and a Gaussian filter (FWHM 8.0 mm), followed by decay correction.

A PET or PET/CT study was carried out prior to the start of CIRT (average 11 ± 7.3 days; 0–34 days) and approximately one month after the completion of the treatment (average 30 ± 9.4 days; 11–69 days). The patients fasted for at least four hours prior to the study. For the emission data, on average, 703 ± 63 MBq (19 ± 1.7 mCi) or 518–884 MBq (14–24 mCi) of MET was administered intravenously, and collection was started after 23 minutes. Regarding the difference in the sensitivity in the PET scanners, static emission scans were performed for 30 min using the ECAT EXACT HR+ and for 15 min by the ECAT EXACT 47 for each bed position, respectively [21,22,28]. For the PET/CT study, prior to a PET emission scan, an unenhanced helical CT scan (tube voltage: 120 kV, tube current range: 10-300 mA) was performed with a pitch of 0.5 s/rotation. CT images were reconstructed with a filtered back projection algorithm (512 × 512 matrix size, and a slice thickness of 2.0 mm) and the μ-map data for attenuation correction were calculated from the CT data. The CT scan was performed from the calvarium to the femoral region, and the PET emission data were collected for these regions. The emission scan was performed for three minutes per bed, and was performed for seven to nine beds. PET and PET/CT images were interpreted in consensus by a nuclear medicine specialist physician (with more than 25 years of experience in nuclear medicine and PET) and two oral and maxillofacial surgeons who were certified as PET nuclear medicine specialists (five and six years of PET experience, respectively).

### Statistical analysis

The survival period was defined as the time period from the start of CIRT until death or completion of this study. The univariate analysis was conducted using a log-rank test that compared the associations among the clinical, radiographic and pathological parameters with the survival curves between two groups. The Cox model was used in a multivariate survival regression analysis, while adjusting the survival comparisons for various factors that otherwise influenced survival. The hazard ratio was calculated from this model.

Tumor accumulation was evaluated using a semi-quantitative TNR (tumor to normal tissue ratio) evaluation, which was calculated using the SUN Ultra 60 and SUN Blade 2500 PET software programs (version 7.22; Siemens CTI, Knoxville, TN, USA), and VOX BASE II (version 2.69j; J-MAC System, Inc, Sapporo, Hokkaido, Japan). The TNR was calculated using the following formula: TNR = [mean counts per pixel of tumor regions of interest]/[mean counts per pixel of normal tissue regions of interest]. Two normal tissue regions of interest (ROIs) were set in the muscles of the posterior region of the neck, and the mean counts for these ROIs were used to calculate the TNR. In some cases, ROIs were difficult to determine by PET, but adding anatomical information obtained by CT or MRI could allow them to be set.

The univariate and multivariate analyses were carried out regarding the TNR prior to treatment (TNRpre), TNR following treatment (TNRpost) and the residual ratio of TNR changes (TNRratio), as well as the age, gender and tumor size (three clinical factors). The univariate analysis was conducted to identify the statistically significant relationships between the TNRs and treatment outcomes, and to determine the optimal cut-off values for dividing the patients into two groups. Then, using the cut-off values derived from the univariate analysis, the multivariate analysis was conducted for the relationships that were found to be statistically significant in the univariate analysis. The TNRratio was calculated as follows; [TNRpre]/[TNRpost] × 100 (%). A difference of *p* < 0.05 was considered to be significant. To define the two groups in the Kaplan-Meier analysis, the most appropriate cut-off levels were determined to be the midpoint of the range, so that the lowest *p* value was obtained in each statistical analysis [29]. Forty millimeters was employed as a cut-off value for the tumor size (peak dimension), because this value is regarded to be the boundary between T2 and T3 tumors of the head and neck in many sites, such as the oral cavity, large salivary gland, oropharynx and hypopharynx [25]. The statistical analyses were conducted using the Statview software program (version 5.0; SAS Institute Inc, Cary, North Carolina, USA) [30].

## Results

### Clinical results and PET imaging

**Figure 1:**
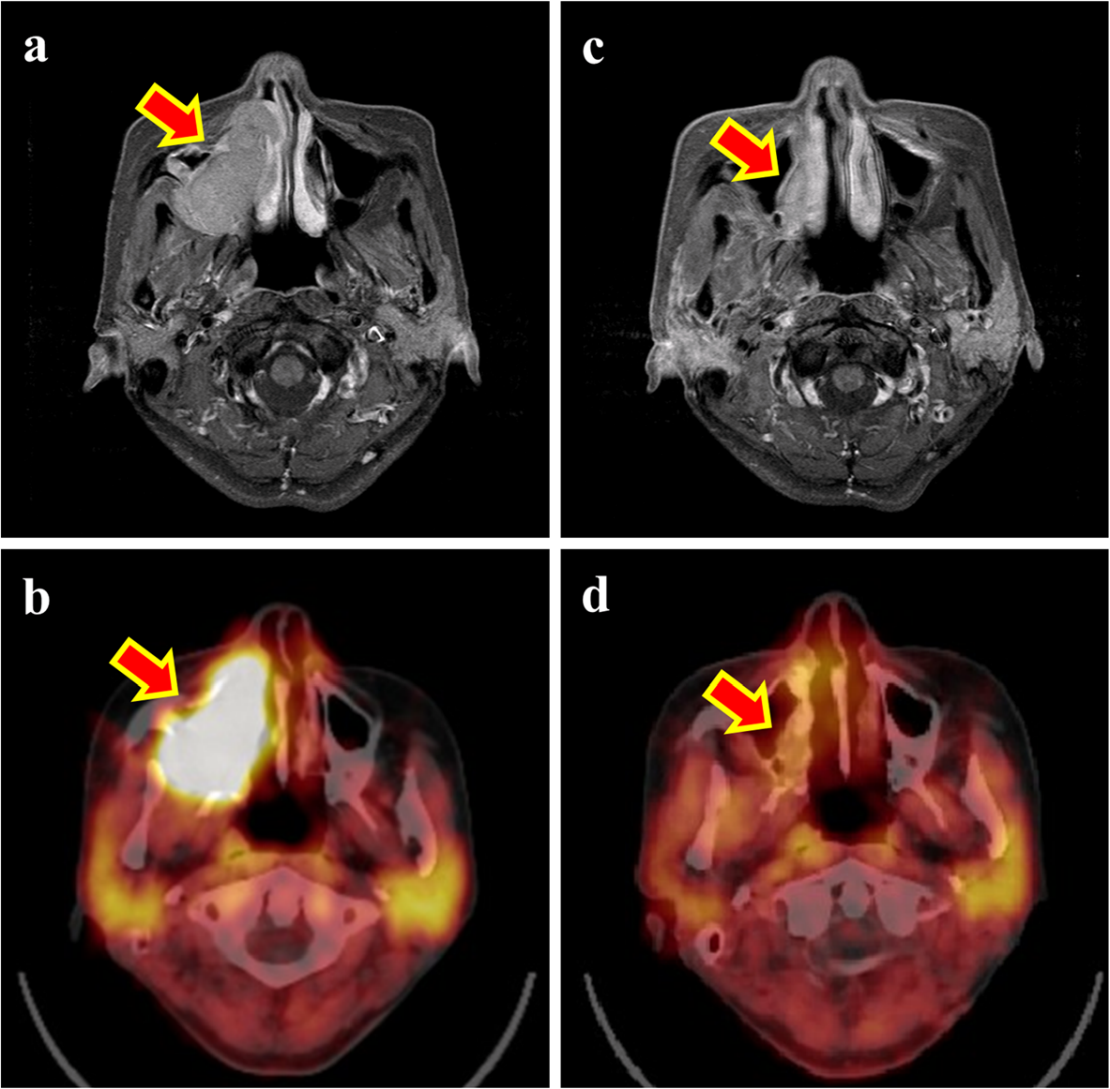
**A 49-year-old female who received 57.6 GyE for paranasal sinus cancer.** The arrows indicate the tumors. **a**. Magnetic resonance imaging (MRI) showed a 53 × 44 mm mass in the paranasal sinus region. **b**. A methionine (MET)-PET/CT image prior to treatment demonstrated a high accumulation mass in the region shown in the MRI. **c**. MRI revealed tumor shrinkage 44 days after carbon ion radiotherapy. **d**. The MET-PET/CT image after the treatment demonstrated decreased MET uptake.

**Figure 2:**
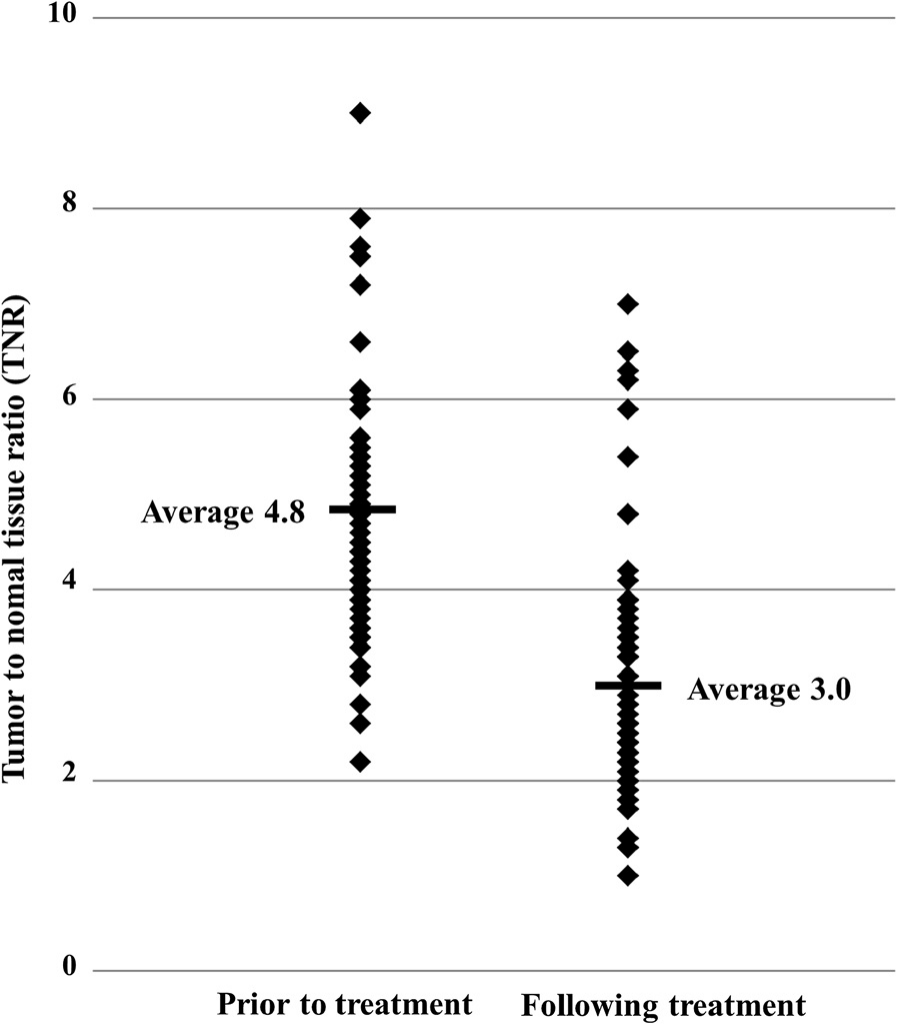
**A comparison of the tumor to normal tissue ratio (TNR) prior to and following CIRT.** The average values of the TNR prior to treatment and after treatment were 4.8 ± 1.5 (2.6-9.0) and 3.0 ± 1.3 (1.0-7.0), respectively, and the difference between them was significant (paired-*t* test, *p* < 0.0001).

Among the 67 total patients, 21 patients (31%) died due to cancer progression (cancer death). Twenty-one patients (31%) and 27 patients (40%) developed local recurrence and metastasis during the follow-up period, respectively. The tumor size (radius of the maximum length) before treatment averaged 49 ± 16 mm (range, 20–87 mm). All adenoid cystic carcinomas of the head and neck were visually distinguishable from the surrounding tissues by the MET-PET and PET/CT studies prior to heavy-particle therapy. In several cases, the tumors were close to the oral cavity mucosa, but their boundaries were relatively difficult to define due to the influence of physiological accumulation, in which introducing anatomical information from CT or MRI could identify the tumors. Representative cases in which CIRT was particularly effective are shown in Figure 1. The average values of the TNRpre, TNRpost, as well as the TNRratio were 4.8 ± 1.5 (2.6-9.0), 3.0 ± 1.3 (1.0-7.0) and 66 ± 26% (19-150%), respectively, demonstrating that there were significant differences in the average values of the TNRpre and TNRpost (paired-*t* test, *p* < 0.0001) [Figure 2]. The five-year disease-specific survival rate was 69%, and the 10-year disease-specific survival rate was 45%.

### Univariate analysis

**Figure 3:**
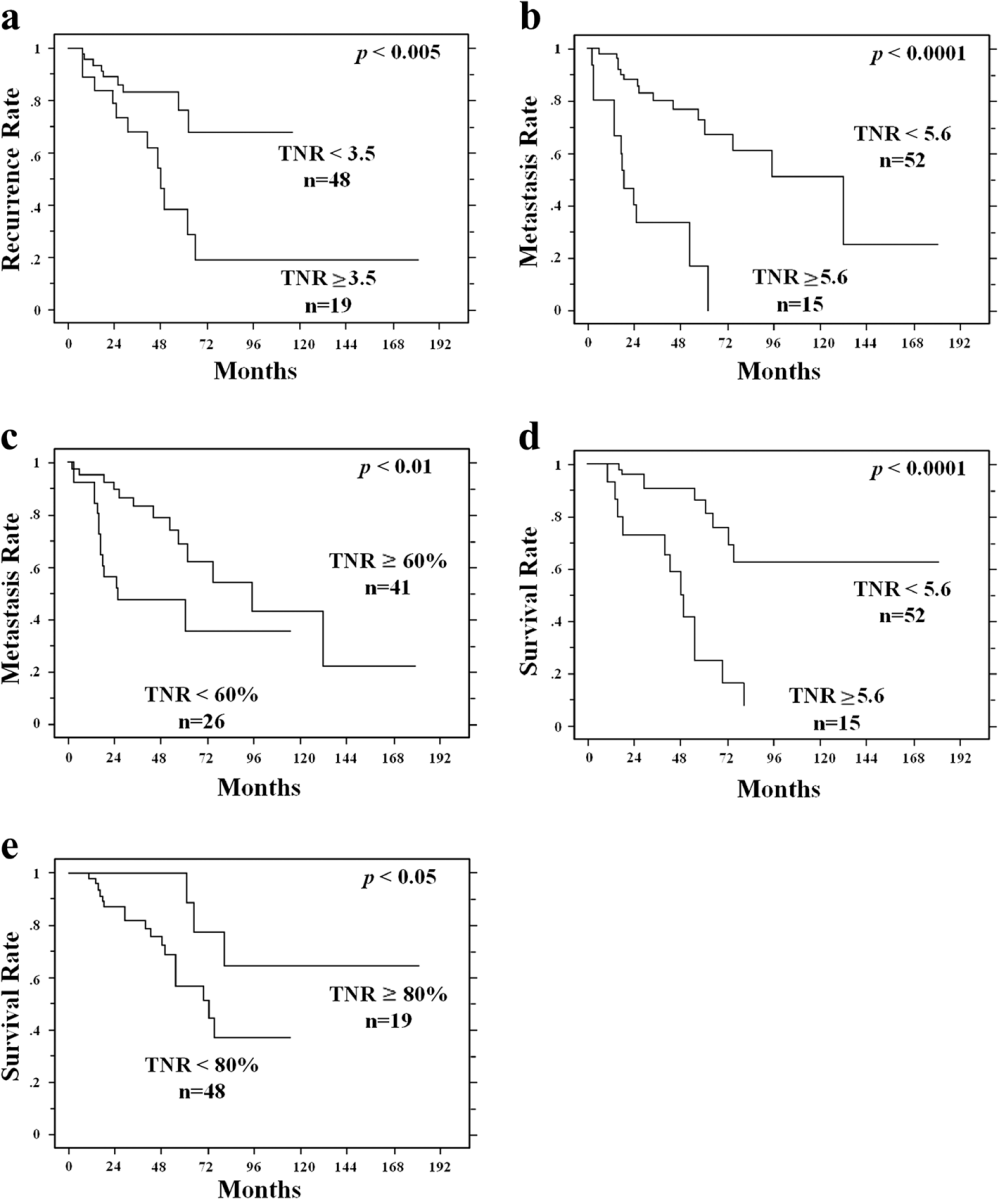
**The results of a univariate analysis using the Kaplan-Meier method. a**. The five-year local recurrence rates demonstrated a significant difference between the two groups divided by the TNR following treatment (the cut-off value was 3.5, *p* < 0.005). **b**. The five-year metastasis rates demonstrated a significant difference between the two groups divided by the TNR prior to treatment (the cut-off value was 5.6, *p* < 0.0001). **c**. The five-year metastasis rates demonstrated a significant difference between the two groups divided by the residual ratio of the TNR (the cut-off value was 60%, *p* < 0.01). **d**. The five-year disease-specific survival rates demonstrated a significant difference between the two groups divided by the TNR prior to treatment (the cut-off value was 5.6, *p* < 0.0001). **e**. The five-year disease-specific survival rates demonstrated a significant difference between the two groups divided by the residual ratio of the TNR (the cut-off value was 80%, *p* < 0.05).

**Figure 4:**
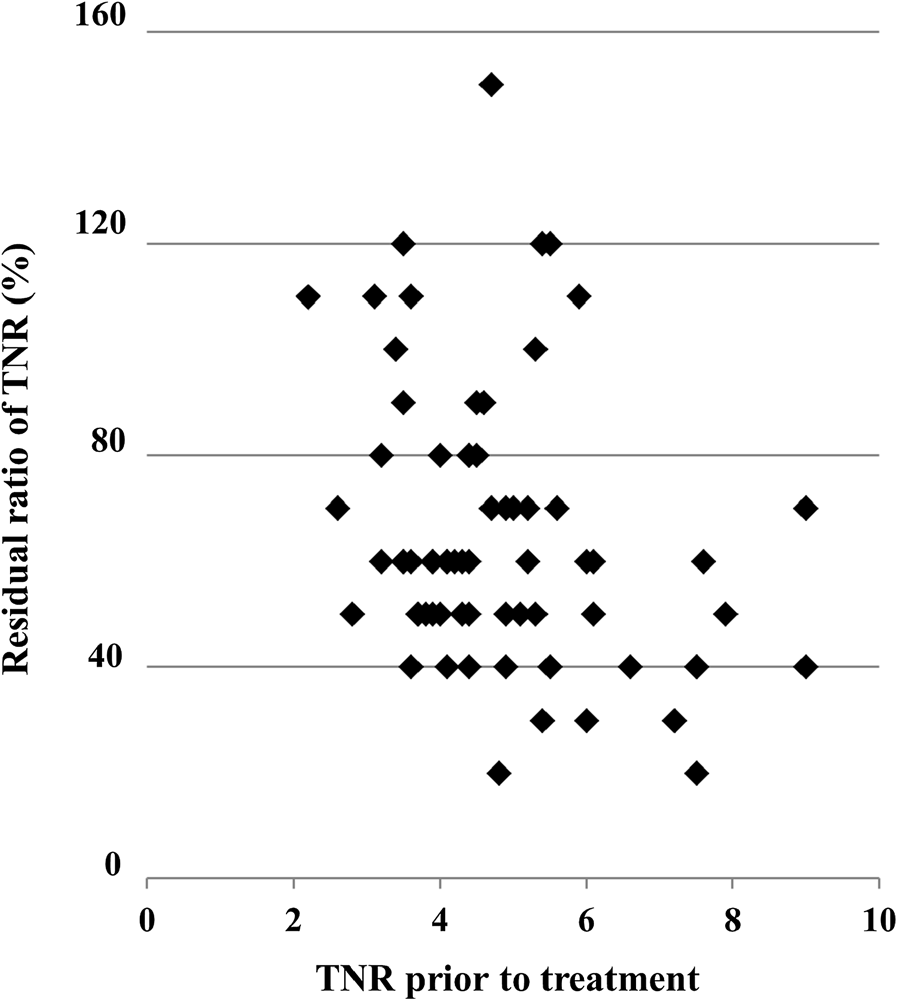
**The correlation between the residual ratio of the TNR and the TNR prior to CIRT.** There was a significant negative correlation observed between the residual ratio of the TNR changes and the TNR prior to treatment (Spearman rank correlation coefficient, ρ = −0.32, *p* < 0.02).

In the univariate analysis, the group with a high TNRpre showed significantly more metastasis compared to the group with a lower value (cut-off value = 5.6, *p* < 0.0001), and also showed a poorer prognosis (disease-specific survival) (cut-off value = 5.6, *p* < 0.0001). Groups with a high TNRpost value showed significantly more local recurrence compared to the group with a lower value (cut-off value = 3.5, *p* < 0.005). Regarding the TNRratio, there were significantly more occurrences of metastasis in the group with a low TNRratio (cut-off value = 60%, *p* < 0.01), and as would be expected, the disease-specific survival was poorer in this group (cut-off value = 80%, *p* < 0.05); however, a significant negative correlation (Spearman rank correlation coefficient, ρ = −0.32, *p* < 0.02) was observed between the TNRratio and the TNRpre, with a tendency for the TNRratio to be lower in cases with a high TNRpre. The above results are summarized in the Table 3 and Figures 3 and 4.

**Table 3:** Results of a univariate analysis

	**Recurrences**	**Metastasis**	**Survival**
TNR prior to treatment (*P* value)	-	≥5.6	≥5.6
		(*p* < 0.0001)^*^	(*p* < 0.0001)^*^
TNR following treatment (*P* value)	≥3.5	‒	‒
	(*p* < 0.005)^*^		
Residual ratio of TNR (*P* value)	‒	<60%	<80%
		(*p* < 0.01)^*^	(*p* < 0.05)^*^

*Significant difference (*p* < 0.05, log-lank).

### Multivariate analysis

A multivariate analysis (Cox proportional hazard model) was conducted using the age, gender, tumor size, TNRpre, TNRpost and TNRratio. Three clinical factors were added to investigate whether or not the TNRpre, TNRpost and TNRratio can be used as predictive factors for local recurrence, metastasis and disease-specific survival. That is, the average age of 54 years old and a tumor size (radius of the maximum length) of 40 mm were used as cut-off values. Regarding the TNRpre, a multivariate analysis was conducted for metastasis and disease-specific survival in which significant differences had been observed in the univariate analysis. A cut-off value of 5.6 was set for each TNRpre. Regarding the TNRpost, a multivariate analysis was conducted for local recurrence, in which significant differences had been observed in the univariate analysis. A cut-off value of 3.5 was set for the TNRpost. Regarding the TNRratio, a multivariate analysis was conducted for metastasis and disease-specific survival, in which significant differences had been observed in the univariate analysis. The cut-off values were set at 60% for metastasis and 80% for disease-specific survival.

Regarding the development of local recurrence in the investigation using the TNRpost and three clinical factors, the TNRpost (*p* < 0.04) and tumor size (*p* < 0.03) were identified as influential factors. In the investigation of metastasis using the TNRpre and three clinical factors, the TNRpre (*p* < 0.002) was the only influential factor. In the investigation of metastasis using the TNRratio and three clinical factors, the TNRratio (*p* < 0.04) and tumor size (*p* < 0.03) were both identified as influential factors.

With regard to the disease-specific survival in the investigation using the TNRpre and three clinical factors, the TNRpre (*p* < 0.02) and age (*p* < 0.03) were identified as influential factors, while in the investigation using the TNRratio and three clinical factors, the tumor size (*p* < 0.04), age (*p* < 0.05) and gender (*p* < 0.02) were all identified as influential factors affecting the disease-specific survival. The above results are summarized in Tables 4, 5 and 6.

**Table 4:** Prognostic factors including prior to treatment in the patients using multivariate analysis

**Variable**	**Multivariate**			
	**CI**	**CI**	***p*value**	**HR**
**Metastasis**				
TNR prior to treatment (≥5.6 vs. <5.6)	0.089	0.557	0.0013	0.222
Tumor size (>40 vs. ≤40)	0.166	1.082	0.0725	0.423
Age (≥54 vs. <54)	0.339	1.827	0.5766	0.787
Gender (male vs. female)	0.342	2.003	0.6743	0.827
**Survival**				
TNR prior to treatment (≥5.6 vs. <5.6)	0.090	0.751	0.0128	0.259
Tumor size (>40 vs. ≤40)	0.129	1.325	0.1373	0.414
Age (≥54 vs. <54)	0.097	0.843	0.0232	0.287
Gender (male vs. female)	0.181	1.700	0.3023	0.555

*CI* confidence interval, *HR* hazard ratio.

**Table 5:** Prognostic factors including following treatment in the patients using multivariate analysis

**Variable**	**Multivariate**			
	**CI**	**CI**	***p*value**	**HR**
**Recurrence**				
TNR following treatment (≥3.5 vs. <3.5)	0.142	0.907	0.0303	0.359
Tumor size (>40 vs. ≤40)	0.092	0.852	0.0249	0.280
Age (≥54 vs. <54)	0.250	1.751	0.4056	0.662
Gender (male vs. female)	0.219	1.387	0.2061	0.552

*CI* confidence interval, *HR* hazard ratio.

**Table 6:** Prognostic factors including the residual ratio of TNR changes prior to and following treatment in the patients using multivariate analysis

**Variable**	**Multivariate**			
	**CI**	**CI**	***p*value**	**HR**
**Metastasis**				
Residual ratio of TNR (≥60% vs. <60%)	1.051	5.229	0.0374	2.344
Tumor size (>40 vs. ≤40)	0.135	0.863	0.0230	0.342
Age (≥54 vs. <54)	0.350	1.889	0.6311	0.814
Gender (male vs. female)	0.222	1.119	0.0914	0.499
**Survival**				
Residual ratio of TNR (≥80% vs. <80%)	0.652	8.254	0.1939	2.319
Tumor size (>40 vs. ≤40)	0.097	0.927	0.0364	0.300
Age (≥54 vs. <54)	0.112	0.980	0.0459	0.332
Gender (male vs. female)	0.113	0.805	0.0166	0.302

*CI* confidence interval, *HR* hazard ratio.

*Residual ratio of TNR*: residual ratio of tracer uptake change prior to and following carbon ion radiotherapy.

## Discussion

Primary adenoid cystic carcinomas of the head and neck are relatively rare tumors [1,2], with slow development, but which have a strong tendency for local invasion, leading a frequency of metastasis and a poor prognosis [2-6]. Currently, surgery and postoperative irradiation have been the most common therapeutic methods used to treat these cancers. According to a review by Dodd *et al.*, the efficacy of chemotherapy is highly variable. Regimens with platinating agents, anthracyclines and alkylating agents have shown the most consistent reaction rates, with efficacy rates ranging from 25-33% [8]. Spiro *et al.* reported that local recurrence was observed in 62% and metastasis in 38% of patients in an investigation of 196 cases of salivary gland primary adenoid cystic carcinomas (surgery only, 188 cases; radiation only, 8 cases) [5]. Khan *et al.* reported the outcomes of various treatments, and the overall rate of local recurrence was 12-52% and the rate of metastasis was 19-52% [3]. In our investigation, local recurrence developed in 31% of the 67 cases and metastasis was observed in 40% of cases; however, considering that 76% of the 67 cases were in stage T4, it should be noted that the local control by CIRT was relatively high compared to that of other therapeutic methods.

It has been reported that the development of local recurrence or metastasis 10 years following treatment is not rare in patients with adenoid cystic carcinomas [31], with one case of lung metastasis among our cases occurring 132 months after treatment. It is believed that strict follow-up is required over a long time period. Fordice *et al.* have reported that, in 160 cases of primary adenoid cystic carcinoma of the head and neck for which surgery alone or surgery/radiation combination therapy were performed, the five-year survival rate was 89% and the 10-year survival rate was 67.4% [32]. A review by Khan *et al.* reported that the five-year survival rate was 67-73% and the 10-year survival rate was 44-51% [3]. In our study, the five-year survival rate was 69% and the 10-year survival rate was 45%, which was approximately the same as those reported in the other studies.

No other report using PET targeting primary adenoid cystic carcinoma of the head and neck has been published. However, Lindholm *et al.* performed MET-PET prior to and following treatment with radiation therapy for cancer of the head and neck in 15 cases (SCC in 13 cases, ACC in one case and plasmocytoma in one case) and reported that the MET accumulation following treatment was significantly related to the histological effect [33]. Chesnay *et al.* showed that MET-PET accumulation after the completion of one course of chemotherapy for hypopharynx squamous cancer was significantly correlated with a reduction in the tumor mass, as measured by MRI at the completion of three courses of chemotherapy. Although there were no significant differences between the groups with a good/poor effect observed by the MET-PET evaluation, there were differences in the two-year survival rates, which were 83% and 57% [34]. Hasebe *et al.* carried out a statistical (univariate and multivariate analysis) investigation regarding the use of MET-PET to predict the therapeutic efficacy of CIRT for 39 head and neck adenocarcinoma patients, and their multivariate analysis revealed that the TNRpre was a significant factor influencing the metastasis and disease-specific survival, while the TNR following the treatment was associated with the local recurrence, metastasis and disease-specific survival, and the change in accumulation was associated with the development of metastasis and the disease-specific survival. Consequently, it was reported that MET-PET allowed for a prediction of the therapeutic efficacy [24]. These references seem to suggest that MET-PET is potentially useful for determining the therapeutic efficacy of radiation therapy and chemotherapy.

As a result of our multivariate analysis, the TNRpost based on the MET accumulation and the tumor size were found to be significantly influential factors in terms of local recurrence. In terms of indices regarding MET accumulation that are associated with the occurrence of metastasis, the TNRpre and TNRratio were identified as the significantly influential factors. With regard to the disease-specific survival, the TNRpre and age were the significantly influential factors, while the tumor size, patient age and gender were also influential in other combinations.

Kokemueller *et al.* pointed out that the tumor size as an important factor for predicting local recurrence [6]. Spiro *et al.* pointed out that the size of the primary focus was an influential factor on metastasis [1]. Jones *et al.* have suggested that the prognosis was significantly better for T1 + T2 patients, with a 10-year survival rate of 80% for T1 + T2, while it was only 30% for T3 + T4 cases [35]. In our results, the tumor size was also a significantly influential factor for local recurrence, in addition to metastasis and disease-specific survival. Moreover, Jones *et al.*[35] reported that males with primary adenoid cystic carcinomas of the head and neck had a significantly better prognosis than females, while our results suggested that the five-year survival rate was 53% for males and 78% for females, indicating that females had a better disease-specific survival in our patient cohort. The TNRpre was a significant factor predicting both the development of metastasis and the disease-specific survival. Similarly, the TNRpost was a significant factor for predicting local recurrence. However, there was a significant negative correlation observed between the TNRratio and TNRpre. That is, cases with a low TNRratio were likely to have a high TNRpre (i.e. negative correlation), and therefore, to have a tendency toward more frequent occurrence of metastasis. It is believed that the TNRratio is less useful than the TNRpre.

In our present investigation, it was suggested that it was possible to predict the effects of CIRT using MET-PET (or PET/CT). Based on these results, it is expected that PET studies will be useful for selecting an optimal individualized treatment strategy, for example, by introducing a strict short-term follow-up or active combination therapy with chemotherapy, etc., when an increased risk of metastasis is identified in cases with a high TNRpre. The determination of the effect of CIRT using MET-PET (or PET/CT) is also expected to contribute to clinical studies of other malignant tumors being carried out at our institute.

## Conclusion

In patients with adenoid cystic carcinomas of the head and neck on which CIRT was performed, MET-PET (or PET/CT) performed prior to treatment could predict the development of future metastasis and the disease-specific survival. MET-PET (or PET/CT) performed following treatment was able to predict the development of local recurrence. Thus, MET-PET or PET/CT is useful for determining the therapeutic efficacy of CIRT.

## Competing interests

The authors declare that they have no competing interests.

## Authors’ contributions

ST and KY, Conception and design of experiment. SO, Statistical analyses and interpretation of data. KT, Performing PET and PET/CT studies. AH, Selection and management of the patients. KK and TS, Drafting and finalizing the article. TK, Approval of the final version. All authors read and approved the final manuscript.

## References

[B1] Spiro RH Huvos AG Strong EW Adenoid cystic carcinoma of salivary origin. A clinicopathologic study of 242 cases Am J Surg 1974 128 4 512 520 10.1016/0002-9610(74)90265-7 4371368

[B2] Kim KH Sung MW Chung PS Rhee CS Park CI Kim WH Adenoid cystic carcinoma of the head and neck Arch Otolaryngol Head Neck Surg 1994 120 7 721 726 10.1001/archotol.1994.01880310027006 8018324

[B3] Khan AJ DiGiovanna MP Ross DA Sasaki CT Carter D Son YH Adenoid cystic carcinoma: a retrospective clinical review Int J Cancer 2001 96 3 149 158 10.1002/ijc.1013 11410883

[B4] Takagi D Fukuda S Furuta Y Yagi K Homma A Nagahashi T Clinical study of adenoid cystic carcinoma of the head and neck Auris Nasus Larynx 2001 28 S99 S102 11683353 10.1016/s0385-8146(01)00073-6

[B5] Spiro RH Distant metastasis in adenoid cystic carcinoma of salivary origin Am J Surg 1997 174 5 495 498 10.1016/S0002-9610(97)00153-0 9374223

[B6] Kokemueller H Eckardt A Brachvogel P Hausamen JE Adenoid cystic carcinoma of the head and neck-a 20 years experience Int J Oral Maxillofac Surg 2004 33 1 25 31 10.1054/ijom.2003.0448 14690656

[B7] Mendenhall WM Morris CG Amdur RJ Werning JW Hinerman RW Villaret DB Radiotherapy alone or combined with surgery for adenoid cystic carcinoma of the head and neck Head Neck 2004 26 2 154 162 10.1002/hed.10380 14762884

[B8] Dodd RL Slevin NJ Salivary gland adenoid cystic carcinoma: a review of chemotherapy and molecular therapies Oral Oncol 2006 42 8 759 769 10.1016/j.oraloncology.2006.01.001 16757203

[B9] Blakely EA Cell inactivation by heavy charged particles Radiat Environ Biophys 1992 31 3 181 196 10.1007/BF01214826 1502327

[B10] Tobias CA Blakely EA Alpen EL Castro JR Ainsworth EJ Curtis SB Molecular and cellular radiobiology of heavy ions Int J Radiat Oncol Biol Phys 1982 8 12 2109 2120 10.1016/0360-3016(82)90554-5 6819271

[B11] Ando K Koike S Ohira C Chen YJ Nojima K Ando S Accelerated reoxygenation of a murine fibrosarcoma after carbon-ion radiation Int J Radiat Biol 1999 75 4 505 512 10.1080/095530099140438 10331856

[B12] Kanai T Endo M Minohara S Miyahara N Koyama-ito H Tomura H Biophysical characteristics of HIMAC clinical irradiation system for heavy-ion radiation therapy Int J Radiat Oncol Biol Phys 1999 44 1 201 210 10.1016/S0360-3016(98)00544-6 10219815

[B13] Hoffman RM Altered methionine metabolism. DNA methylation and oncogene expression in carcinogenesis. A review and synthesis Biochim Biophys Acta 1984 738 1–2 49 87 6204687 10.1016/0304-419x(84)90019-2

[B14] Hoffman RM Unbalanced transmethylation and the perturbation of the differentiated state leading to cancer Bioessays 1990 12 4 163 166 10.1002/bies.950120404 2185747

[B15] Stern PH Hoffman RM Elevated overall rates of transmethylation in cell lines from diverse human tumors In Vitro 1984 20 8 663 670 10.1007/BF02619617 6500606

[B16] Stern PH Wallace CD Hoffman RM Altered methionine metabolism occurs in all members of a set of diverse human tumor cell lines J Cell Physiol 1984 119 1 29 34 10.1002/jcp.1041190106 6707100

[B17] Wheatley DN On the problem of linear incorporation of amino acids into cell protein Experientia 1982 38 7 818 820 10.1007/BF01972291 7106252

[B18] Mizoe JE Tsujii H Kamada T Matsuoka Y Tsuji H Osaka Y Dose escalation study of carbon ion radiotherapy for locally advanced head-and-neck cancer Int J Radiat Oncol Biol Phys 2004 60 2 358 364 10.1016/j.ijrobp.2004.02.067 15380567

[B19] Tsujii H Mizoe JE Kamada T Baba M Tsuji H Kato H Clinical results of carbon ion radiotherapy at NIRS J Radiat Res 2007 48 Suppl A A1 A13 17513896 10.1269/jrr.48.a1

[B20] Okada T Kamada T Tsuji H Mizoe JE Baba M Kato S Carbon ion radiotherapy: clinical experiences at National Institute of Radiological Science (NIRS) J Radiat Res 2010 51 4 355 364 10.1269/jrr.10016 20508375

[B21] Zhang H Yoshikawa K Tamura K Tomemori T Sagou K Tian M (11)C]methionine positron emission tomography and survival in patients with bone and soft tissue sarcomas treated by carbon ion radiotherapy Clin Cancer Res 2004 10 5 1764 1772 10.1158/1078-0432.CCR-0190-3 15014030

[B22] Koizumi M Saga T Yoshikawa K Suzuki K Yamada S Hasebe M C-11-methionine-PET for evaluation of carbon ion radiotherapy in patients with pelvic recurrence of rectal cancer Mol Imaging Biol 2008 10 6 374 380 10.1007/s11307-008-0156-1 18679757

[B23] Tamura K Yoshikawa K Ishikawa H Hasebe M Tsuji H Yanagi T Carbon-11-methionine PET imaging of choroidal melanoma and the time course after carbon ion beam radiotherapy Anticancer Res 2009 29 5 1507 1514 19443358

[B24] Hasebe M Yoshikawa K Ohashi S Toubaru S Kawaguchi K Sato J A study on the prognostic evaluation of carbon ion radiotherapy for head and neck adenocarcinoma with C-11 methionine PET Mol Imaging Biol 2010 12 5 554 562 10.1007/s11307-010-0318-9 20369300

[B25] Hermanek P Sobin LH International Union Against Cancer TNM classification of malignant tumors: fourth, fully revised edition 1987 Geneva: Published by International Union Against Cancer 13 35

[B26] World Health Organization WHO Handbook for Reporting the Results of Cancer Treatment, Definitions of objective response 1979 Geneva: World Health Organization 23 25 48

[B27] Långström B Antoni G Gullberg P Halldin C Malmborg P Någren K Synthesis of L- and D-[methyl-11C]methionine J Nucl Med 1987 28 6 1037 1040 3585494

[B28] Zhang H Yoshikawa K Tamura K Sagou K Tian M Suhara T Carbon-11-methionine positron emission tomography imaging of chordoma Skeletal Radiol 2004 33 9 524 530 15483754 10.1007/s00256-004-0815-5

[B29] Fiorino C Sanguineti G Cozzarini C Fellin G Foppiano F Menegotti L Rectal dose-volume constraints in high-dose radiotherapy of localized prostate cancer Int J Radiat Oncol Biol Phys 2003 57 4 953 962 10.1016/S0360-3016(03)00665-5 14575825

[B30] Landau S Rabe-Hesketh S StatView for windows, version 5.0 Stat Methods Med Res 1999 8 4 337 341 10730337 10.1177/096228029900800411

[B31] Ganly I Patel SG Coleman M Ghossein R Carlson D Shah JP Malignant minor salivary gland tumors of the larynx Arch Otolaryngol Head Neck Surg 2006 132 7 767 770 10.1001/archotol.132.7.767 16847187

[B32] Fordice J Kershaw C El-Naggar A Goepfert H Adenoid cystic carcinoma of the head and neck: predictors of morbidity and mortality Arch Otolaryngol Head Neck Surg 1999 125 2 149 152 10037280 10.1001/archotol.125.2.149

[B33] Lindholm P Leskinen-Kallio S Grénman R Lehikoinen P Någren K Teräs M Evaluation of response to radiotherapy in head and neck cancer by positron emission tomography and [11C]methionine Int J Radiat Oncol Biol Phys 1995 32 3 787 794 10.1016/0360-3016(95)00007-L 7790265

[B34] Chesnay E Babin E Constans JM Agostini D Bequignon A Regeasse A Early response to chemotherapy in hypopharyngeal cancer: assessment with (11)C-methionine PET, correlation with morphologic response, and clinical outcome J Nucl Med 2003 44 4 526 532 12679395

[B35] Jones AS Hamilton JW Rowley H Husband D Helliwell TR Adenoid cystic carcinoma of the head and neck Clin Otolaryngol Allied Sci 1997 22 5 434 443 10.1046/j.1365-2273.1997.00041.x 9372255

